# Relationship of tumor marker CA125 and ovarian tumor stem cells: preliminary identification

**DOI:** 10.1186/s13048-015-0132-8

**Published:** 2015-03-28

**Authors:** Hui Zhang, Yongan Yang, Yifeng Wang, Xinping Gao, Weiming Wang, Hui Liu, Haipeng He, Yijuan Liang, Kun Pan, Hongli Wu, Junrong Shi, Huiling Xue, Ling Liang, Zhihuci Cai, Yanfang Fan, Yanyan Zhang

**Affiliations:** Affiliated Hospital of HeBei University, Baoding City, HeBei Province China; The Third Hospital of Baoding/Baoding Cancer Hospital, Baoding City, HeBei Province China; Department of Obstetrics and Gynecology, Third Affiliated Hospital, Guangzhou Medical College, Guangdong Province, China; Department of Epidemiology and Health Statistic, College of Public Health of Hebe University, Baoding City, HeBei Province China; Department of Obstetrics and Gynecology, Qingyuan County Hospital, Baoding City, HeBei Province China; Department of Obstetrics and Gynecology, Wangdu County Hospital, Baoding City, HeBei Province China

**Keywords:** Ovarian cancer, CA125, Tumor stem cell

## Abstract

**Purpose:**

The purpose of this study is to identify a prospective association between CA125 and tumorigenic ovarian cancer cells, using the new method of orthotopic transplantation (1).

**Method:**

After making the surgical ovarian cancer specimen into cell suspension, we separated the tumorigenic cells from the nontumorigenic cancer cells based on cell surface marker (cancer antigen CA125 and lineage markers) expression. We developed a SCID mice model in which the CA125+/ lineage- and CA125-/ lineage- cells were injected into ovarian parenchyma by use of a microinjector. As a measure of effectiveness of tumor-forming, tumor weight, abdominal distension, ascites volume and activity, subcutaneous fat were determined or observed. Immunohistochemistry was done to determine tumor cell markers.

**Results:**

We found that the cells of CA125+/ lineage- were able to form new tumors; whereas, an equal quantity of CA125-/lineage- cells failed to form any tumors. The new generated tumor contained additional CA125-/lineage- tumorigenic cells as well as the phenotypically diverse population of nontumorigenic cells. Quantities were judged to be significantly different *P* < 0.0001.

**Conclusion:**

CA125+/ lineage- cells, which may be ovarian cancer stem cells, were the source for tumor recurrence. The strategies designed to target this cell population may lead to more effective therapies.

Ovarian cancer is the fifth most common cause of cancer death in women and is the most common cause of death by a gynecological tumor [1], where the recurrence and drug resistance are the focus and difficulty of the treatment. As early as 1977, Hamburger et al. found that only a portion of the ovarian cancer cells may be cultured in vitro or in vivo to generate colonies, and they proposed that tumor stem cells might be found in the solid tumors [2]. These cells are not sensitive to chemotherapy and radiotherapy and are the “seed cells” for tumor recurrence and metastasis. This is the main target of our therapy. However, due to lack of information about the abnormal expression spectra of membrane protein of the tumor stem cells and the phenotype of normal stem cells in ovarian cancer, one can only isolate and identify the ovarian cancer stem cells by screening stem cell markers associated with the epithelial stem cells that lie on the surface of the ovarian cells.

In this study, our goal was to apply the orthotopic transplantation mode to preliminarily identify an association between the tumor marker CA125 and ovarian tumor stem cells [3]. Tumor antigen CA125 is a classical marker for early diagnosis and recurrence of ovarian cancer [4,5], where its value is positively correlated with the degree of tumor malignancy and the possibility of recurrence. Our experiment’s design was based on the phenotypic stability of the tumor stem cells [6]. Using flow cytometry, the primary cancer cells were divided into CA125+/lineage-group and CA125-/lineage-group. The in vivo tumorigenicity of these two groups were observed and satisfactory results were obtained.

## Materials and methods

### Materials

#### Mice

A total of 40 female SCID (Severe combined immunodeficiency) mice of 6 to 8 weeks old was purchased from Shanghai SLRC Laboratory Animal Center (NO. 0014393 and NO. 0015117). Their weight ranged from 15 g to 18 g. 1 week later, they received intraperitoneal injection of Etoposide (10 mg/kg dose, diluted into 200 ml with HBSS, Hank’s balanced salt solution), and meanwhile received a small piece of Estrogen sustained release tablet in the hypoderm of the neck, which was placed through trocar and was to lower the immunity and shorten the period of neoplasia. And 6 days later, they were inoculated with cell suspension under 4% Chloral Hydrate (0.1 ml/g dose) anesthesia. The animal experiments were approved by the animal care and use committee in HeBei University.

#### Cells

Human ovarian cancer specimens were obtained within 1 hour of surgical resection (frozen disease results in epithelial ovarian carcinoma) and placed in cellular suspension under aseptic conditions. Patients were diagnosed as ovarian cancer by preoperative CT scan. This study was approved by the ethics committee and patients signed informed consent.

#### Reagents

The first antibody OC125 (ab1107) was purchased from Abcam company. The second antibody labeled by allophycocyanin (APC) was purchased from Unitech Company. Anti-CD140b labeled by phycoerythrin (PE phycoerythrin 28D4) was purchased from American Pharmingen Company. The Fluorescein isothiocyanate (FITC)-labeled anti-CD31 (11–0319) and FITC-labeled anti-mouse class I major histocompatibility complex (MHC) antibody (11–5998, H-2Kd/H-2Dd) were purchased from American eBioscience Company. FITC-labeled lineage-specific antibodies (340546; CD3, CD16, CD19, CD20, CD14, CD56) were purchased from American BD Company. Fetal bovine serum, trypsin and EDTA were purchased from American GIBCO Company. And the Metrigel was purchased from American BD Biosciences Company. PI dye was awarded by Institute of Pharmaceutical Technology of the First Affiliated Hospital of Guangzhou Medical College, and other analytical reagents were purchased from Guangzhou WeiJia Technology Limited.

#### Instruments

Flow cell sorter (BECKMAN) (Beckman Coulter, Inc. American), Flow cytometry (for cells detection of BD FACS Ariat™) (BD Biosciences, American), Centrifuge machine (Anke TDL-40B), superspeed refrigerated centrifuge and confocal microscopy (LSM 510META) (ZEISS, German) and fluorescence microscopy (Axioplanz, ZEISS, German).

### Methods

#### Mice preparation

A total of 40 SCID mice were raised in the SPF (specific pathogen free) laboratory of Zhongshan Ophthalmic Hospital Center and the animal occupancy permit number was SKXY Guangdong 2005–0058. Mice were anesthetized with 4% chloral hydrate (0.1 mL/1 g).

#### Preparation of vaccinated primary tumor cell suspension

The fresh human ovarian cancer specimens were cut into small blocks under aseptic conditions and applied with pancreatin containing 0.02% ethylene diamine tetra-acetic acid (EDTA) and incubated at 37°C for 15–20 min, while blown with a 10 mL straw every 3–4 min to mix the tissue blocks and pancreatin thoroughly. After that DMEM containing 20% fetal bovine serum was added to stop digestion, the cells were rinsed twice with HBSS, and filtered with a 200 wells filter. Next, the tissues were collected and applied with mixture of HBSS and matrigel (1:1).

#### Antibody labeling and flow sorting

After conducting cell counting, cells were placed into a 5 mL tube, and rinsed with HBSS containing 2% heat inactivated calf serum(HICS)3 times; each rinse lasted for 5 min with a centrifugal speed of 350 g at 4°C and light-proof. Then applied with 100 uL HBSS (10^6^ cells/100 μL) containing 2% HICS. First antibodies such as PI, lineage-specific antibodies, CD140b, CD31, and OC125 were added with a dilute concentration of 10^6^cells/2 μL, incubated at 4°C and light-proof conditions for 1 h, rinsed with HBSS containing 2% HICS twice, followed by suspension with 100 μL HBSS/2% HICS (10^6^ cells /100 μL) and applied with 1–4 μL second antibody (anti-OC125) and incubated at 4°C and light-proof conditions for another 1 h, rinsed with HBSS/2% HICS twice and suspended with 1 mL HBSS/2% HICS. Antibodies, including first the antibodies OC125 and APC-labeled (red) second antibody, were used for labeling CA125 tumor cells, and PE-labeled (orange color) anti-CD140b was used to eliminate normal epithelial cells. FITC-labeled (green light) anti-CD31 was used to eliminate blood platelets, myelomonocytic and lymphocytes. The FITC-labeled lineage-specific antibodies included anti-CD2, −CD3 -CD10, −CD16, −CD14, −CD18, −CD19, −CD64. Antibodies with unspecified manufacturer were purchased from PharMingen Company and they were marked directly or indirectly with different fluorescence to facilitate the sorting. Among the lineage-specific antibodies (anti-CD2, −CD3 -CD10, −CD16, −CD14, −CD18, −CD19, −CD64, -CD140b, CD31), CD2 and CD3 were used for inhibiting T-lymphocytes reaction, CD10 and CD19 for B-lymphocytes, CD16 for NK cells, CD14 for monocyte, CD18 for granulocyte, CD31 for blood platelet, myelomonocyte and lymphocyte, CD64 for neutrophil granulocyte, CD140b for human normal fibroblasts, smooth muscle cells, glial cells, and chondrocytes. PI (Spontaneous red fluorescence, can be isolated from the APC) was used to label dead cells. A flow cell sorter (BECKMAN) was used for cell sorting, where forward scatting and side scattering were applied to remove overlapping cells. In the sorting process, cells labeled by green, orange, and spontaneous fluorescence were removed, while the red labeled cells were expressions of the CA125+/lineage-ovarian cancer cells and the remaining were CA125-/lineage-ovarian cancer cells.

#### Animal vaccination

**Figure 1:**
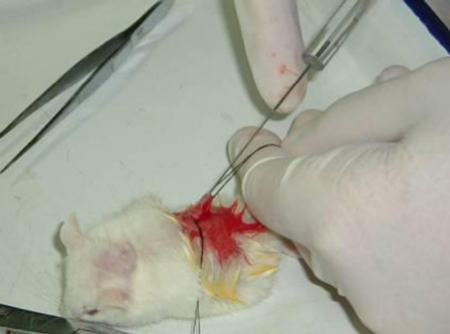
**Data when mice were injected with cell suspension.**

A total of 1.52 × 10^5^ CA125+/lineage-cells and 11.1 × 10^5^ CA125-/lineage-cells were obtained. Negative cells were conducted with negative gradient dilution, of which 1.52 × 10^5^ cells were selected and the rest were discarded, and the selected cells were re-suspended with fetal bovine serum/Matrige (1:1). A total of 40 SCID mice was randomly divided into 4 groups: positive group (A and B groups), negative group (A and B groups), experimental control group and blank control group, each containing 10 mice. The positive group was vaccinated with CA125+/lineager-cells, of which A group (5 mice) and B group (5 mice) were vaccinated with 2 × 10^4^ and 1 × 10^4^ cells, respectively, with a vaccination dose of 10 μL each. The experimental control group was vaccinated with10 μL fetal bovine serum (1:1) and nothing was applied in the blank control group. All the mice were raised under the same conditions. The vaccination methods referred to reference [3], and were illustrated in Figure 1.

#### Animal observation

**Figure 2:**
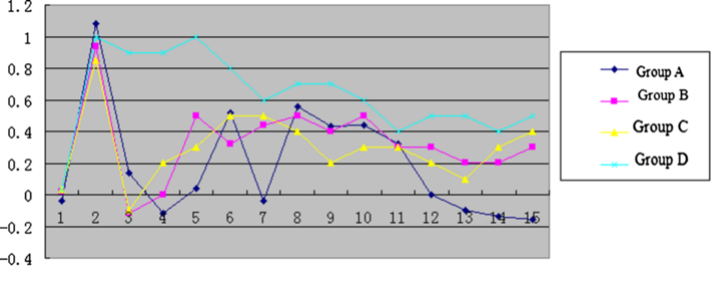
**The growth curve of four groups of mice weight.**

Each mouse was labeled with a random number according to a random table prior to vaccination and weighted every week. Every week, palpation was conducted in mice at the vaccination site, and the reduction of subcutaneous fat, touchable abdominal mass, abdominal bloating, rounded protuberance, activity reduction and other signs associated with tumor development were visually observed. About 3 months later, mice began to become thin and appeared to have reduced their physical activity. Six months later, the mice were executed by breaking their necks; bilateral ovaries and related organs were harvested and their abdominal cavities were dissected for to determine metastasis. The weight growth curve of mice are shown in Figure 2. Group A was positive group. Group B was negative group. Group C was the experimental control group. Group D was the control group. Group A B C curves first increased and then decreased, especially the curve of group A decreased significantly in the 5^th^ month. Group D curve had been a growing trend.

#### Pathology test

After 6-months of observation, the vaccinated ovary and the contralateral ovary of each mouse were cut into 2 parts, of which one part was fixed with 4% neutral formaldehyde liquid, followed by paraffin-embedded, general slice and H&E staining, as well as immunohistochemistry for detecting the CA125 expression (first antibody dilution: 1:50, second antibody dilution: 1:100, second antibody was the mouse monoclonal antibody against human ovarian cancer antigen (CA125), and OC125). The other part conducted with frozen section and labeled with fluorescent antibody: indirect APC-labeled mouse anti-human CA125, and FITC-labeled anti-mouse I type MHC molecular, or H-2K^d^/H-2D^d^ (first antibody dilution: 1:50, second antibody dilution: 1:100, second antibody was the mouse monoclonal antibody against human ovarian cancer antigen (CA125) and OC125 without nucleus staining). A confocal laser scanning microscopy was used to observe the staining results and electronic microscopy was used to observe the morphology of tumor cells.

#### Statistical analysis

SAS software was used for statistical analysis. Single variance analysis and LSD/*t* test was used to calculate P values within each group and between the groups, where *P* < 0.0001 was considered statistically significant.

## Results

### Sorting results by flow cytometry

**Figure 3:**
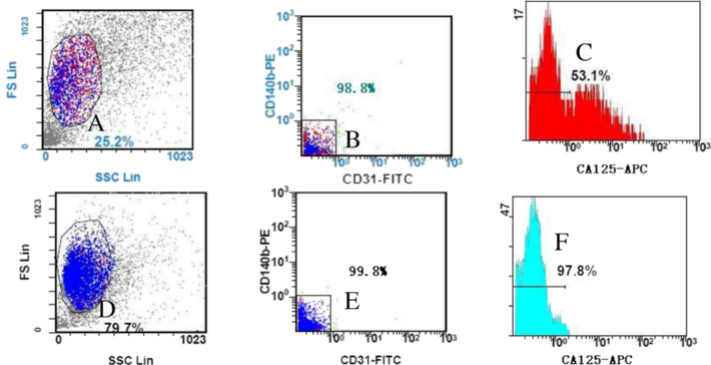
**The flow cytometry test results of primary tumor cells after different fluorescent antibody labeling.** The window of **A** contained live tumor cells to be sorted, and cells in **B** window were obtained from **A** window, which were FITC-negative and PE-negative cells. Cells in window of **C** were obtained from **B** window, where cells inside the window or on the left of the horizontal line were FITC-, PE- and CA125- negative cells, while cells outside the window or on the right of the horizontal line were FITC-negative, PE-negative and CA125-positive cells. Plots in **D**, **E** and **F** were testing results of sorted negative cells. Compared with **A** window, dead cells and cell debris in **D** window significantly reduced. Cells in **E** window were obtained from **D** window, which detected the purity of FITC- and PE-negative cells, while cells in **F** window were obtained from **D** window, which detected purity of FITC-, PE- and CA125-negative cells.

In Figure 3, the window of A contained live tumor cells to be sorted, and cells in B window were obtained from A window, which were FITC-negative and PE-negative cells. Cells in window of C were obtained from B window, where cells inside the window or on the left of the horizontal line were FITC-, PE- and CA125- negative cells, while cells outside the window or on the right of the horizontal line were FITC-negative, PE-negative and CA125-positive cells. Plots in D, E and F were testing results of sorted negative cells. Compared with A window, dead cells and cell debris in D window significantly reduced. Cells in E window were obtained from D window, which detected the purity of FITC- and PE-negative cells, while cells in F window were obtained from D window, which detected purity of FITC-, PE- and CA125-negative cells.

### Tumorigenesis of vaccinated ovary

**Figure 4:**
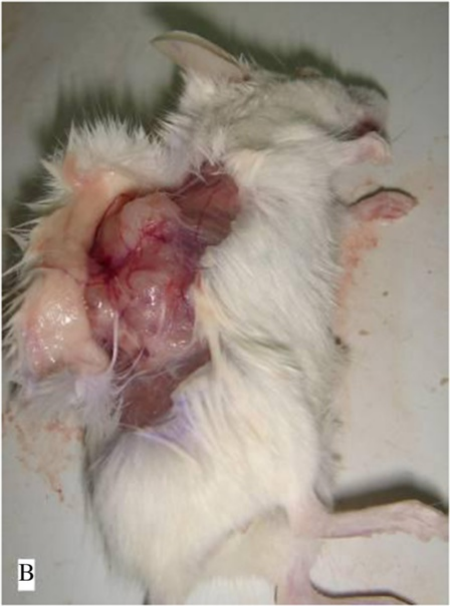
**The state after anatomy after tumor formation.**

**Figure 5:**
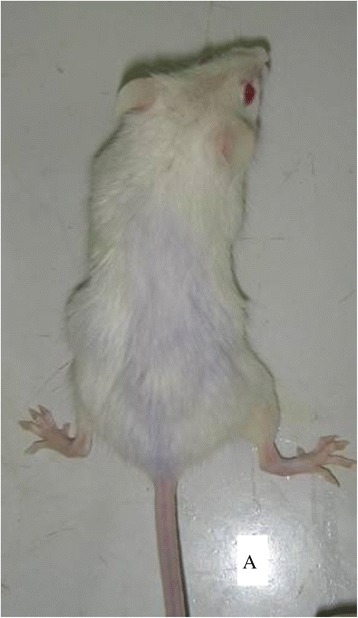
**The state of pre-anatomy after tumor formation.**

**Figure 6:**
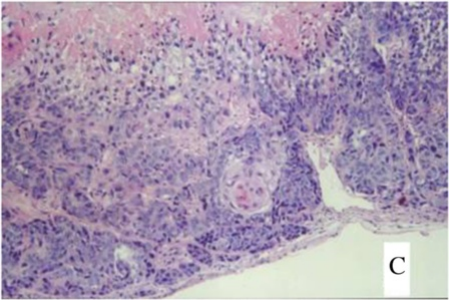
**The ovarian tumor on H&E staining of the vaccinated tumor cells (×40).**

**Figure 7:**
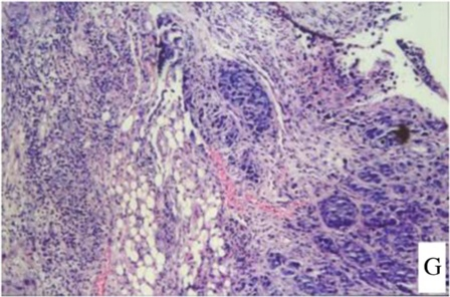
**A case had peritoneal metastasis.**

**Figure 8:**
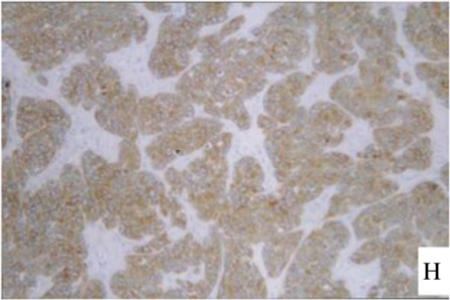
**The immunohistochemical staining of the ovarian tumor for detecting expression of CA125 (brown color).**

**Figure 9:**
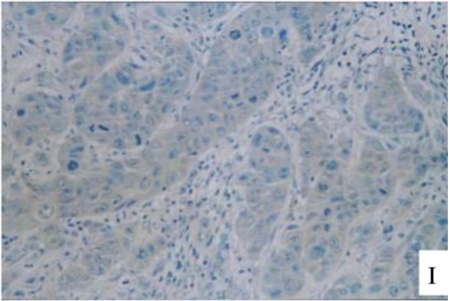
**The immunohistochemical staining of the ovarian tumor for detecting expression of CA125 (brown color).**

**Figure 10:**
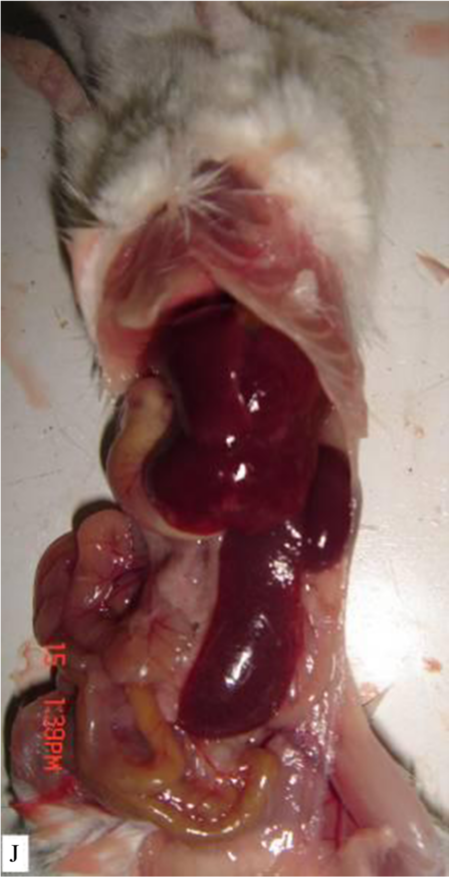
**Liver metastasis.**

**Figure 11:**
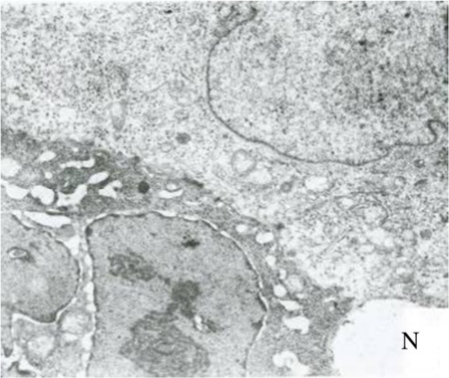
**The SEM results of liver metastases.**

**Figure 12:**
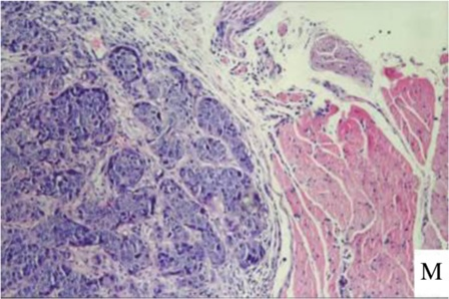
**The pelvic muscle metastasis.**

**Figure 13:**
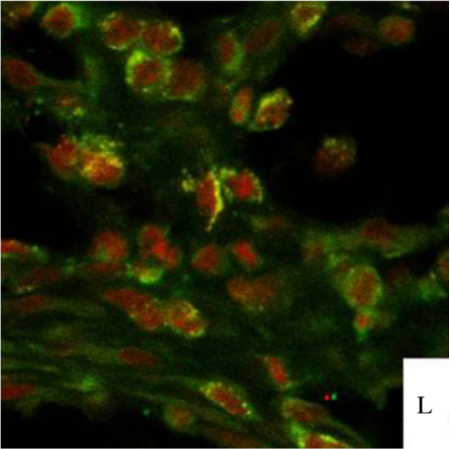
**The expressions of human CA125 (red light) and mouse antigen (green light) of the ovarian tumor.**

As seen in data presented in Table 1, 8 tumors were found in the positive group. As shown in the follow Figures, no tumor was observed in the negative and control groups. Tumorigenic status were showed in Figures 4, 5. Figure 4 is the state of pre-anatomy after tumor formation, Figure 5 is the state after anatomy. Figure 6 illustrated the ovarian tumor on H&E staining of the vaccinated tumor cells (×40). One case had peritoneal metastasis (Figure 7). Figures 8 and 9 showed the immunohistochemical staining of the ovarian tumor for detecting expression of CA125 (brown color). Two cases had liver metastasis, as shown in Figures 10 and 11. Figure 11 was the tumor transferred to liver under the electron microscopy, where a tumor cell was at the upper right, and a solid nucleus liver cell was found at the left bottom. Two cases had pelvic muscle metastasis, (Figure 12). Figure 13 showed the expressions of human CA125 (red light) and mouse antigen (green light) of the ovarian tumor. Analysis by SAS FREQ showed that the difference among the positive groups with different CA125+ /lineage-magnitude was not significant, P=0.4444. The difference between CA125+ /lineage-group and CA125- /lineage-group was statistically significant, (P<0.0001) While the difference between CA125+ /lineage-group and the control group (blank control and control groups) was significant (P<0.0001), and the difference between CA125-/lineage-group and the control group was not significant.

**Table 1:** **Tumor number and vaccination number in study mice**

**Un-treated**	**2 × 10** ^**4**^ **/10 μl**	**1 × 10** ^**4**^ **/10 μl**	**10 μl HBSS/Matrige**	**Blank**
**Experimental group**	**A group**	**B group**		
CA125+/lineage-	3/5	5/5		
CA125-/lineage	0/5	0/5		
**Control group**				
Control group			0/10	
Blank control group				0/10

## Discussion

Ovarian cancer is characterized by insidious onset, rapid progression, and lack of effective early diagnosis; approximately 70% patients are diagnosed in the advanced stages of the disease, and when remission is achieved, there is a high postoperative recurrence rate. Though significant progress of surgery and chemotherapy has been achieved in recent years, the 5-year survival rate in advanced ovarian cancer is still hovering at 20% to 30%. Efforts have been focused on finding new and more effective means to improve the prognosis for patients diagnosed with ovarian cancer.

Theory of tumor stem cell believes that tumor stem cells account for 0.01% ~ 0.1% of the total number of tumor cells with unlimited proliferative potential. They can proliferate by means of symmetric and asymmetric splits, so as to generate new tumor stem cells, initial tumor cells, initial tumor stem cells, and tumor precursor cells, etc. [7,8]. The current methods of treating tumors are focused on most cells in the tumor tissue, rather than only the tumor stem cells. Therefore, though the tumor tissues fade after surgical resection or chemotherapy, the remaining stem cells will generate tumors again, resulting in tumor recurrence and metastasis. Thus, finding the specific biological characteristics of tumor stem cells may enable targeting or selective killing tumor stem cells, prevent tumor recurrence, and metastasis.

Traditionally, it was believed that ovarian cancer was caused by menstruation-induced periodic destruction of epithelial tissues resulting in tumorigenesis. Recent pathological studies have found that many ovarian cancers occur in the distal oviduct and even in the injured location of the endometria [9,10]. However, the above method is disputed since the exact origin of ovarian cancer is still unclear as yet. Ovarian tumor stem cells are believed to be the root cause of ovarian cancer recurrence. However, specific markers of ovarian tumor stem cells have not been unified. Currently, common known markers are CD133, Nestin, CD24, CD166 and CD44 [11-13]. However, due to lack of information about the abnormal expression spectra of membrane protein of the tumor stem cells and phenotype of normal stem cells in ovarian cancer, one can only isolate and identify the ovarian cancer stem cells by screening cell markers associated with the epithelial stem cells on the ovarian surface. Treatment of the selective targeting stem cells is still in its infant stage, since it is still unclear how many markers there are for tumor stem cells [14]. It is necessary to find out more specific markers and explore their physiological functions, thus to achieve better understanding of the differentiation process from multipotent stem cells to tissues at different stages, and applied these as a new targeting anti-cancer therapy [15,16].

CA125, found in 1981, is an inhomogeneous, high molecular weight mucin-like glucoprotein. Bast [17,18] applied ovarian serous cystadenocarcinoma serial OVCA443 to BALB/c mice and purified with myeloma to obtain a monoclonal antibody, named OC125, and its corresponding antigen was named CA125. CA125 is located in the cavity epithelial cells during the embryonic development process, and disappears in the first few hours after birth, but appears again in ovarian cancer cells [19,20]. In recent years, many studies have been conducted to investigate the relation of CA125 quality in the body fluids and its occurrence, clinical staging, and histological type of systemic malignant tumors, as well as lymph node metastasis. It has been found that [21] the involving risk of ovarian cancer in healthy population with serum CA125 > 30 U/mL was significantly increased and it was positively correlated with the concentration in the blood. For menopause women without symptoms, the incidence of CA125 elevation to ovarian cancer significantly increases. With the progress of ovarian cancer, increase in clinical stages and the occurrence of tumor metastasis (disease aggravating), the serum CA125 concentration gradually increases which is positively correlated with number of tumor cells. In patients with effective surgical treatment and chemotherapy, the CA125 quickly decrease. In cases of recurrence and drug resistance, the CA125 elevation appears prior to the clinical symptoms, indicating CA125 is an early marker for recurrence and drug resistance, which is similar to the characteristics of the tumor stem cells. However, no reports have been found to reveal the relationships between them. Is the CA125 another marker for ovarian tumor stem cells? In this study, we aimed to explore the relationship between CA125 and ovarian tumor stem cells, and applied CA125 as a surface marker, then sorted tumor stem cells quickly and effectively by using the flow sorting method.

Studies have found that tumor stem cells have phenotypic stability, which means in addition to differentiating into most of the mature tumor cells; they can also generate new tumor stem cells, which owns the same markers as the primary tumor stem cells and is the source of tumor recurrence and metastasis [22,6]. This experiment was designed based on its phenotypic stability. Ovarian cancer cells were sorted according to different expressions of negative and positive CA125 by using a flow cytometry, and then cells were orthotopically implanted into the ovary of SCID mice. We observed the tumorigenesis of tumor cells with different markers; the tumor cells were digested and suspended, followed by being labeled with antibodies and undergoing mouse passage for observing tumorigenicity, so as to confirm the relation of ovarian tumor stem cells and CA125 and to find their specific markers. This study provides theoretical and experimental basis for specifically killing ovarian tumor stem cells and preventing tumor recurrence.

In this experiment, we preliminary identified the relationship between tumor marker CA125 and ovarian cancer stem cells, and obtained satisfactory results The second antibody labeled by monoclonal antibody of CA125 (OC125) and APC was used to isolate tumor cells with CA125 expression, and lineage-specific antibodies labeled by PE, FITC and other fluorescence were used to eliminate human normal cells in the tumor tissues; PI was used to eliminate dead cells. Accurate sorting can be obtained by using flow cytometry since APC, FITC, PE, and PI are 4 different colors of fluorescence. The tumor cells without human normal cells and dead cells were divided into a negative cell group and a positive cell group according to CA125 expression, and they were orthotopically implanted into ovaries of SCID mice with same cell magnitude to compare tumorigenicity of different tumor cells. We found that there were 8 tumors in the positive groups, while there were no tumors in the negative and control groups; the difference was statically significant, indicating that tumor marker CA125 may be one of the marker for ovarian tumor stem cells. Further confirmations will be the focus of our future studies by increasing magnitude in the number of subjects, as well as adding additional tumor markers. Finding the tumor marker for ovarian cancer prompts the targeting or selective killing these “seed cells”, and enables tumor cure, preventing recurrence and metastasis of tumors.

In 1981, Robert et al. [18] found antigen CA125 in the ovarian cancer cells by using monoclonal antibody OC125. Since then, CA125 has been an auxiliary diagnostic marker for ovarian cancer, and research about immunological treatment of ovarian cancer using the antibody’s specificity for CA125 is also emerging. Our experiments showed that CA125 may be one of the surface markers of tumor stem cells, and further studies are needed to investigate the existence of other markers, which is the direction of our future studies. Finding the markers of ovarian cancer stem cells and targeting killing these “seed cells”, making the prevention and treatment of ovarian cancer possible.
